# The Clinical Impact of Proton Pump Inhibitors When Co-Administered With Dual Antiplatelet Therapy in Patients Having Acute Myocardial Infarction With Low Risk of Gastrointestinal Bleeding: Insights From the China Acute Myocardial Infarction Registry

**DOI:** 10.3389/fcvm.2021.685072

**Published:** 2021-09-22

**Authors:** Wence Shi, Lin Ni, Jingang Yang, Xiaoxue Fan, Mei Yu, Hongmei Yang, Mengyue Yu, Yuejin Yang, China Acute Myocardial Infarction (CAMI) Registry study group

**Affiliations:** ^1^Department of Cardiology, Medical Research and Biometrics Center, State Key Laboratory of Cardiovascular Disease, Fuwai Hospital, National Center for Cardiovascular Diseases, Chinese Academy of Medical Sciences and Peking Union Medical College, Beijing, China; ^2^Medical Research and Biometrics Center, State Key Laboratory of Cardiovascular Disease, Fuwai Hospital, National Center for Cardiovascular Diseases, Chinese Academy of Medical Sciences and Peking Union Medical College, Beijing, China; ^3^Langfang People's Hospital, Hebei, China; ^4^First Hospital of Qinhuangdao, Qinhuangdao, China

**Keywords:** proton pump inhibitors, acute myocardial infarction, gastrointestinal bleeding (GIB), co-medication, lower risk

## Abstract

**Background:** The latest guidelines recommend the use of proton pump inhibitors (PPIs) to minimize gastrointestinal bleeding (GIB) in patients receiving dual antiplatelet therapy (DAPT), even though this co-administration may increase the risk of ischemia due to drug interactions. We have noticed that there are few studies conducted on patients with a lower risk of GIB. Therefore, we investigated the clinical effect of co-administration of PPI on DAPT patients with low GIB risk.

**Methods and Results:** From January 2013 to September 2014, a total of 17,274 consecutive patients on DAPT from 108 hospitals with low risk for GIB in the China Acute Myocardial Infarction (CAMI) registry were analyzed. The primary endpoints were GIB and major adverse cardiovascular and cerebrovascular events (MACCE). Multivariate logistic regression analysis and Cox proportional hazard models were used to assess the effect of PPIs use. Of the analyzed patients, 66.6% (*n* = 11,487) were treated with PPIs. PPI use did not show an extra gastrointestinal protective effect in patients with low risk for GIB who were hospitalized and on follow-up after 2 years. Moreover, it was associated with an increased risk of stroke during the 2-year follow-up [hazard ratio (HR) 2.072, 95% confidence interval (CI) 1.388–3.091, *p* = 0.0003] and an increased risk of MI after 6 months (HR 1.580, 95% CI 1.102–2.265, *p* = 0.0119). We found the same results after propensity score matching.

**Conclusion:** PPI use is prevalent in DAPT patients with low GIB risk. PPIs did not show an extra gastrointestinal protective effect, while an increased risk of stroke was observed during the 2-year follow-up.

**Clinical Trial Registration:**www.clinicaltrials.gov, identifier NCT01874691.

## Introduction

Dual antiplatelet therapy (DAPT), a combination of aspirin and an inhibitor of platelet P2Y_12_ receptor, is the most clarified medicine in cardiovascular disease, which is widely recommended in the latest guidelines (1, 2). However, it could cause an increased risk of gastrointestinal bleeding (GIB) (3) and other adverse clinical outcomes (4). Although randomized controlled trials have demonstrated that proton pump inhibitors (PPIs) reduce the rate of recurrent GIB (5), especially in high-risk patients [advanced age (>75); concurrent use of anticoagulants, steroids, or non-steroidals; and *Helicobacter pylori* infection] (6), a potential drug interaction has limited its common use in acute myocardial infarction (AMI) patients (1, 7). We noticed that the impact of PPIs on clinical outcomes when co-administered with DAPT was inconsistent in different studies (8), and the over-prescription of PPIs was increasingly becoming a public health concern (9, 10). Therefore, we investigated the impact of PPIs–DAPT co-medication in patients with low GIB risk and hope to provide more evidence for clinical decisions.

## Methods

### Data Collection

All patients analyzed in our research were from the China Acute Myocardial Infarction (CAMI) registry, which is a prospective, nationwide, multicenter observational study of patients with AMI. The registry includes three levels of hospitals (provincial-, prefectural-, and county-level hospitals, representing typical Chinese governmental and administrative models) covering all provinces and municipalities across mainland China. The CAMI registry was registered with ClinicalTrials.gov (NCT01874691), and this project was approved by the institutional review board central committee at Fuwai Hospital, NCCD of China. All patient data were protected at all times. Detailed descriptions about data management and quality control can be found in the methodological article about the CAMI registry published previously (11).

Simply, all elements (especially outcomes events) are collected, validated, and submitted through a secure, password-protected, web-based electronic data capture system (http://www.CAMIRegistry.org) by the local investigators at each participating site. Trained clinical investigators were employed to ensure the accuracy and reliability of data. Element definitions are accessible to investigators automatically at the point of data entry. The front page of the electronic case report form (eCRF) must be filled out and submitted online within 24 h from patient admission who meet the inclusion criteria.

### Patient Population and Exclusion Criteria

**Figure 1:**
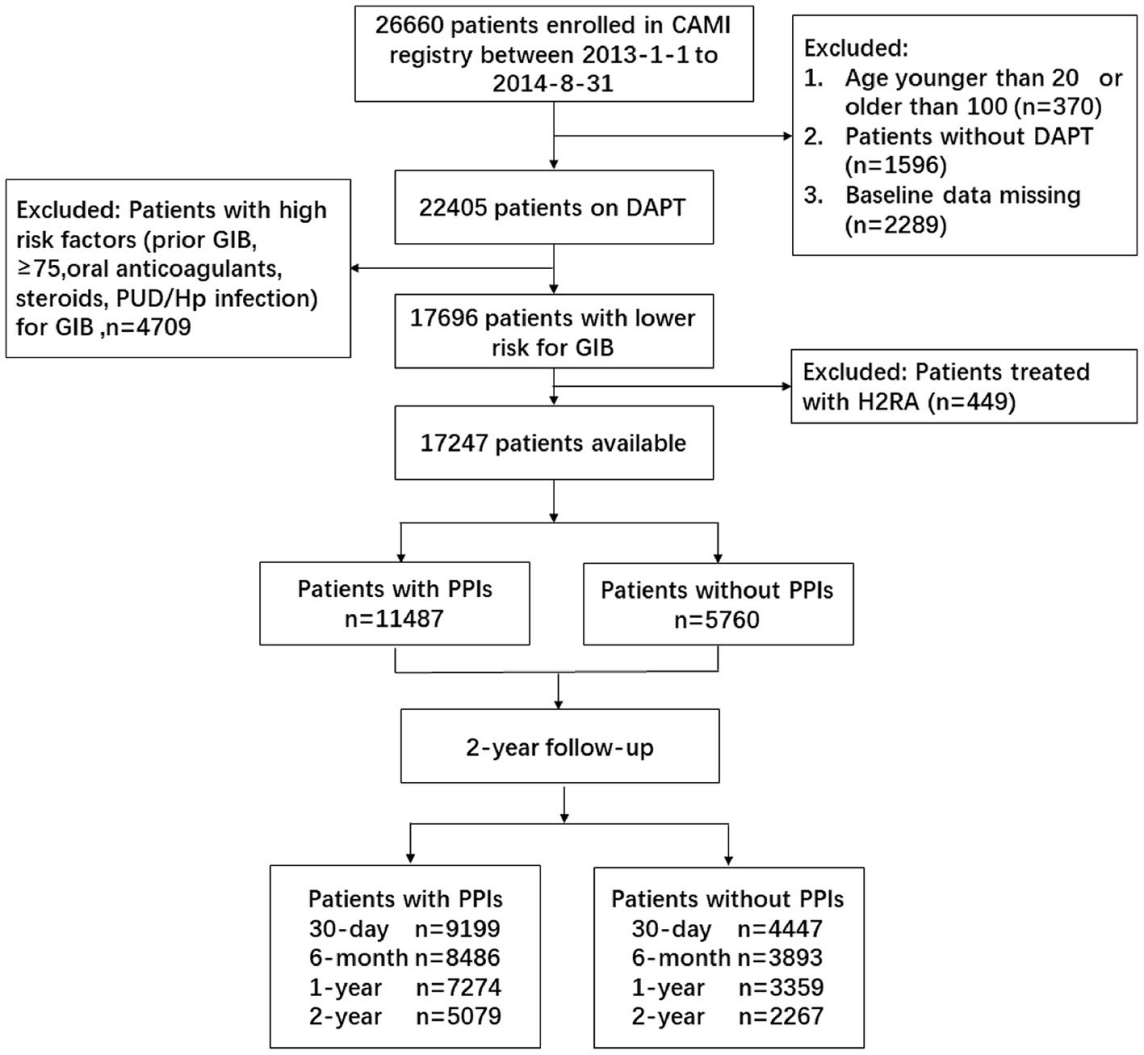
Patient flowchart for the study cohort. CAMI, China Acute Myocardial Infarction; DAPT, dual antiplatelet therapy; GIB, gastrointestinal bleeding; PUD, peptic ulcer disease; PPIs, proton pump inhibitors.

Overall, 26,660 AMI patients from 108 hospitals were enrolled from January 1, 2013 to August 31, 2014. A total of 22,405 patients on DAPT were available, after excluding those with incorrect age (*n* = 370), no DAPT (*n* = 1,596), and missing baseline data (*n* = 2,289). We further excluded 449 patients treated with H2 receptor antagonists for gastrointestinal prophylaxis. A total of 4,709 patients were identified as a high-risk group for GIB [advanced age (>75); concurrent use of anticoagulants, steroids, or non-steroidals; and *H. pylori* infection] according to the guideline (1, 6). The patients with low risk for GIB were identified, excluding the high-risk population, and the data of 17,247 DAPT patients were finally analyzed (Figure 1).

### Outcomes

The primary endpoints were GIB and major adverse cardiovascular and cerebrovascular events (MACCE). GIB was defined as clinically evident GIB (gross hematemesis, heme positive coffee-ground emesis, and heme positive melena). MACCE was a composite endpoint of all-cause death, MI, and stroke. Secondary endpoints were all-cause death, MI, and stroke. All the information is collected using the standardized set of variables and standard definitions that were validated by trained investigators. All variables were coded with CDISC, ICD-10, MedDra, and WHO-DD to make them standardized.

### Patient Follow-Up

Post-discharge study follow-up was conducted *via* centralized telephone interviews by trained personnel at 30 days, 6 months, 1 year, and 2 years. The clinical events were validated by source documents. PPI use was identified at the study baseline and each study follow-up. Patients were excluded if they had quit their PPI use during the follow-up.

### Statistical Analysis

Continuous variables are expressed as mean ± standard deviation or median (25th and 75th percentiles), and categorical variables are presented as percentages. Differences in baseline characteristics and outcomes in patients with and without PPIs were assessed using the chi-square test, Fisher's exact test for categorical variables and analysis of variance test, or the Wilcoxon rank test for continuous variables. Multivariate logistic regression analyses were conducted to evaluate the adjusted effect of PPI use on clinical outcomes. The 2-year follow-up endpoints were modeled using the Cox proportional hazard regression. Clinical characteristics that were imbalanced at a nominal 5% significance level between the two groups, treated or not treated with PPIs, were identified and included in the final adjusted model; other important factors that can affect the clinical endpoints were also included in the final model, although their differences were not significant between the two groups in the univariate analysis (such as the history of diabetes and congestive heart failure). These included age, clinical presentation, and medical therapy (detailed variables included are presented below the relevant tables). Odds ratio (OR) and hazard ratio (HR) were presented with the 95% confidence intervals (CIs). All statistical analyses were performed using SAS version 9.4, and a two-tailed *p* < 0.05 was considered statistically significant.

We also performed propensity score matching (PSM) to select two comparable patients with balanced observed variables. A propensity score was estimated for each patient using a logistic regression model. Patients were matched on estimated propensity scores, with replacement, using the nearest neighbor approach. The detailed information about the propensity score model can be found in the Supplementary Material.

## Results

### Baseline Characteristics

Among 17,247 DAPT patients with low risk for GIB, 66.6% (*n* = 11,487) were treated with PPIs. Patients on PPIs tended to be older (58.37 vs. 57.84, *p* = 0.0042), female (21.8 vs. 20.4%, *p* = 0.0351), and with a higher Killip class (IV 3.4 vs. 2.7%, *p* < 0.0001) and hematocrit (Hct; 41.44 vs. 39.76, *p* = 0.0029) at admission with a history of hypertension (49.6 vs. 46.7%, *p* = 0.0003), MI (8.9 vs. 7.1%, *p* = 0.0273), stroke (8.3 vs. 6.8%, *p* = 0.0007), and malignancy (1.0 vs. 0.7%, *p* = 0.0331). On hospitalization, they were often treated with a GPIIb/IIIa receptor inhibitor (37.0 vs. 26.4%, *p* < 0.0001) and heparin (94.2 vs. 89.7%, *p* < 0.0001). Detailed information on demographic and clinical characteristics is presented in Table 1.

**Table 1:** Baseline clinical data in patients with and without PPIs.

**Variables**	**With PPIs (*n* = 11,487)**	**Without PPIs (*n* = 5,760)**	***P***
**Demographics**			
Age	58.37 ± 11.36	57.84 ± 11.64	0.0042
Female	2,507 (21.8%)	1,177 (20.4%)	0.0351
**Medical history**			
Hypertension	5,696 (49.6%)	2,690 (46.7%)	0.0003
Dyslipidemia	841 (7.3%)	376 (6.5%)	0.0535
Diabetes mellitus	2,286 (19.9%)	1,106 (19.2%)	0.0647
Myocardial infarction	1,022 (8.9%)	409 (7.1%)	0.0273
PCI	514 (4.5%)	259 (4.5%)	0.9477
CABG	37 (0.3%)	19 (0.3%)	0.9328
Congestive heart failure	154 (1.3%)	73 (1.3%)	0.6895
Stroke	952 (8.3%)	394 (6.8%)	0.0007
Chronic kidney disease	104 (0.9%)	54 (0.9%)	0.8349
Malignancy	114 (1.0%)	39 (0.7%)	0.0331
**Admission features**			
STEMI	9,035 (78.7%)	4,324 (75.1%)	<0.0001
Heart rate (beats/min)	78.69 ± 20.90	78.05 ± 18.81	0.6087
Systolic BP (mmHg)	128.37 ± 24.85	129.58 ± 25.49	0.0030
Killip class IV	396 (3.4%)	157 (2.7%)	<0.0001
Hb (g/L)	138.68 ± 19.52	139.16 ± 21.08	0.1455
Hct (%) (Q1:Q3)	41.44 ± 57.01	39.76 ± 14.62	0.0029
CRUSADE score	17.62 ± 13.72	17.97 ± 14.10	0.1184
**Pre-hospital medications**, ***n*****(%)**			
Aspirin	1,079 (9.4%)	605 (10.5%)	0.0212
P2Y_12_ receptor inhibitor	347 (3.0%)	200 (3.5%)	0.1132
**In-hospital medications**, ***n*****(%)**			
**P2Y**_**12**_ **receptor inhibitor**			
Clopidogrel	11,210 (97.59%)	5,536 (96.11%)	0.0002
Ticagrelor	266 (2.32%)	132 (2.29%)	0.0712
GPIIb/IIIa receptor inhibitor	4,255 (37.0%)	1,522 (26.4%)	<0.0001
Oral anticoagulants	93 (0.8%)	142 (2.5%)	<0.0001
Heparin/LMWH	10,816 (94.2%)	5,164 (89.7%)	<0.0001
Statin	3,625 (31.6%)	1,642 (28.5%)	<0.0001
β-Blockers	8,534 (74.3%)	4,067 (70.6%)	<0.0001
ACEI/ARB	4,313 (37.5%)	2,272 (39.4%)	0.0157
**Treatment**, ***n*****(%)**			
Primary PCI	4,181 (36.4%)	2,142 (37.2%)	0.0515
Primary CABG	34 (0.3%)	12 (0.2%)	0.5334
Thrombolysis	816 (7.1%)	426 (7.4%)	0.1121

*PCI, percutaneous coronary intervention; CABG, coronary artery bypass grafting; STEMI, ST-elevation myocardial infarction; BP, blood pressure; Hb, hemoglobin; Hct, hematocrit; CRUSADE, Can Rapid risk stratification of Unstable angina patients Suppress ADverse outcomes with Early implementation of the ACC/AHA guidelines; GPIIb/IIIa, glycoprotein IIb/IIIa; LMWH, low molecular weight heparin; ACEI/ARB, angiotensin converting enzyme inhibitor/angiotensin receptor blocker*.

### In-Hospital Clinical Outcomes

We did not find a protective effect of PPIs against GIB. Another primary efficacy endpoint (composite of all-cause death, MI, and stroke) was similar between patients with PPIs and without PPI use (5.0 vs. 4.7%, adjusted OR 1.026, 95% CI 0.877–1.203, *p* = 0.7189) (Table 2). Results were consistent across all-cause death and MI as presented in Table 2. Furthermore, PPI use was associated with an increased risk for stroke compared with patients without PPI use (0.6 vs. 0.3%, adjusted OR 2.125, 95% CI 1.216–3.682, *p* = 0.0062) (Table 2).

**Table 2:** In-hospital endpoints incidence and adjusted OR among DAPT patients.

**Clinical endpoint**	**With PPIs, (%)**	**Without PPIs, (%)**	***P*-value**	**Adjusted OR (95% CI)**	***P*-value**
GIB	122 (1.1%)	10 (0.2%)	<0.0001	5.574 (2.902-10.697)	<0.0001
**MACCE**	568 (5.0%)	271 (4.7%)	0.4794	1.026 (0.877-1.203)	0.7189
All-cause death	471 (4.1%)	248 (4.3%)	0.6362	0.938 (0.791-1.112)	0.4615
MI	61 (0.5%)	16 (0.3%)	0.0140	1.529 (0.872-2.678)	0.1417
Stroke	69 (0.6%)	16 (0.3%)	0.0026	**2.125 (1.216-3.682)**	**0.0062**

*GIB, gastrointestinal bleeding; MACCE, major adverse cardiovascular and cerebrovascular events; MI, myocardial infarction; OR, odds ratio*.

*Variables included in the model: age; female, hypertension; diabetes mellitus, congestive heart failure, dyslipidemia; stroke; malignancy; STEMI; systolic BP; Killip class IV; Hct; Aspirin; GPIIb/IIIa receptor inhibitor; oral anticoagulants; heparin/LMWH; statin; β-blockers; ACEI/ARB*.

*Bold values indicates statistical difference*.

### Two-Year Follow-Up Results

**Figure 2:**
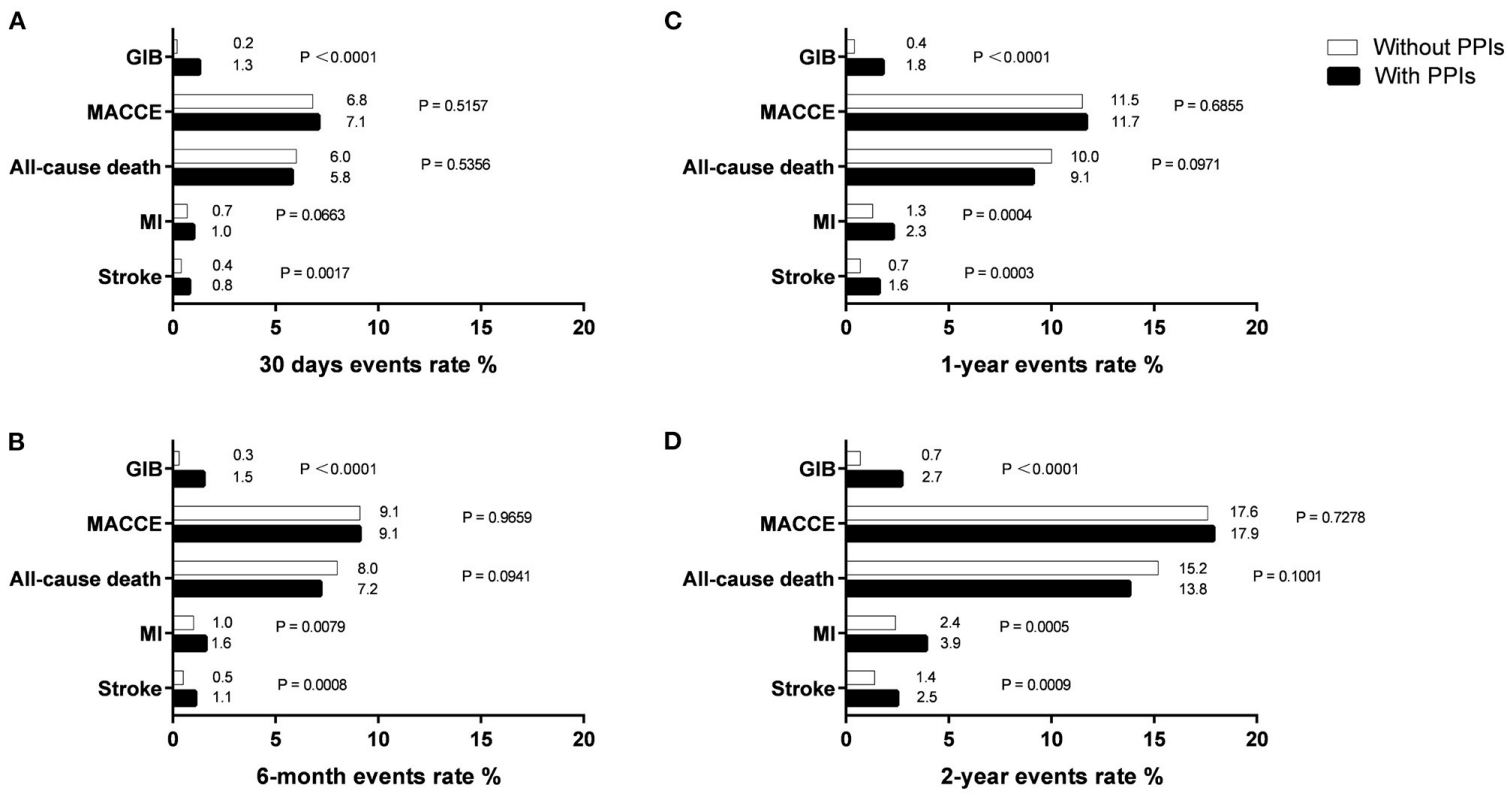
Endpoints events rate at 30 days **(A)**, 6 months **(B)**, 1 year **(C)**, and 2 years **(D)** in patients with and without PPIs.

Patients were followed up throughout a period of 2 years, and event rates at 30 days, 6 months, 1 year, and 2 years are presented in Figure 2. The mean follow-up for patients finally analyzed in our study was 447.7 days with 57.41% lost-to-follow-up. As for MACCE and all-cause death, we found no difference between patients with and without PPIs during follow-up (Table 3). At 6 months, the risk for all-cause death increased significantly in patients treated with PPIs, and the increased risk was seen consistently across all follow-ups in the PPI group. Moreover, PPI co-administration was associated with stroke events for all follow-up points (Table 3).

**Table 3:** 30-Day, 6-month, 1-year, and 2-year adjusted HR of patients with and without PPIs.

**Clinical endpoints**	**30-day**		**6-month**		**1-year**		**2-year**	
	**Adjusted HR (95% CI)**	***P*-value**	**Adjusted HR (95% CI)**	***P*-value**	**Adjusted HR (95% CI)**	***P*-value**	**Adjusted HR (95% CI)**	***P*-value**
GIB	5.577 (2.903-10.696)	<0.0001	4.988 (2.742-9.069)	<0.0001	4.331 (2.488-7.563)	<0.0001	3.650 (2.188-6.082)	<0.0001
**MACCE**	1.049 (0.905-1.211)	0.5024	1.070 (0.934-1.225)	0.3143	1.095 (0.963-1.246)	0.1581	1.123 (0.994-1.270)	0.0588
All-cause death	0.965 (0.822-1.131)	0.6883	0.945 (0.817-1.096)	0.4812	0.940 (0.817-1.083)	0.4110	0.971 (0.848-1.112)	0.6848
MI	1.297 (0.855-1.968)	0.2204	**1.580 (1.102-2.265)**	**0.0119**	**1.812 (1.296-2.536)**	**0.0003**	**1.773 (1.301-2.412)**	**0.0002**
Stroke	**2.202 (1.287-3.763)**	**0.0042**	**2.270 (1.401-3.675)**	**0.0004**	**2.261 (1.454-3.515)**	**0.0002**	**2.072 (1.388-3.091)**	**0.0003**

*GIB, gastrointestinal bleeding; MACCE, major adverse cardiovascular and cerebrovascular events; MI, myocardial infarction; HR, hazard ratio*.

*Variables included in the model: age; female, hypertension; diabetes mellitus, congestive heart failure, dyslipidemia; stroke; malignancy; STEMI; systolic BP; Killip class IV; Hct; Aspirin; GPIIb/IIIa receptor inhibitor; oral anticoagulants; heparin/LMWH; statin; β-blockers; ACEI/ARB*.

*Bold values indicates statistical difference*.

### PSM Results

After PSM, 5,014 patients with PPIs had an estimated propensity score that matched to 5,014 patients without PPI use. PPIs did not show an extra gastrointestinal protective effect, while an increased risk for stroke was seen during the 2-year follow-up (Supplementary Material).

## Discussion

The main findings of our research were as follows: (1) among the DAPT population, 66% of patients with low GIB risk were treated with PPIs. (2) We did not find an extra protective effect of PPIs on the gastrointestinal tract among DAPT patients with low GIB risk. (3) PPI use was associated with an increased risk of stroke in hospital and during the 2-year follow-up and an increased risk of MI after 6 months. PSM did not change the final results.

In our study, PPI use was common in patients with low risk for GIB. We noticed that PPI over-prescription was becoming a new concern in the field of AMI patient management (9, 10). And our former research also emphasized this in the Chinese AMI population (12). Although our study focused on patients with low GIB risk, the PPI use rate (66%) was still higher than that in the ADAPT-DES study (13) (31.4%), PRODIGY trial (14) (37.4%), and TRANSLATE-ACS study (15) (18.2%). This unexpected finding indicated a lower threshold for prescribing PPIs in China, while clinical practice recommendations made by guidelines were better followed in the US (16). This should attract the attention of physicians and administrative personnel to limit over-prescription, which can help reduce the burden on personal costs and the healthcare system.

Randomized controlled trials have demonstrated that PPIs reduce the rate of recurrent GIB in high-risk patients receiving aspirin (5), while few researchers have investigated the effect of PPI use on low-risk patients. Our results indicated that low-risk patients might not benefit from this gastrointestinal prophylaxis. Moreover, the latest clinical guidelines recommended that PPIs co-administration is applicable for minimizing bleeding while on DAPT. However, pharmacokinetic studies showed a potential drug interaction between PPIs and P2Y_12_ receptor inhibitors, which could decrease the effect of DAPT. Both clopidogrel and PPIs require bio-transformation into active metabolite *via* cytochrome P-450 (CYP) enzymes in the liver (17, 18), and physicians raised concerns that competitive inhibition would attenuate its antiplatelet effect, which would increase the risk of ischemic events. However, there were no consistent results in clinical research regarding the effect of co-administration (8, 14, 15). We hypothesized that the benefit of co-administration would be less than the adverse outcomes in patients with low GIB risk.

During hospitalization and the 2-year follow-up, we found an increased risk of stroke in patients with PPI use; few studies have reported the same finding previously. Stroke is more prevalent in the Chinese population (19, 20). Therefore the effect of adverse drug interaction was amplified in patients with low GIB risk. Moreover, we noticed that the adverse effects of PPIs on MI occurred after 6 months. This indicated that PPI use could help improve DAPT compliance within 6 months and patients could benefit from this gastrointestinal prophylaxis. This result was similar to another research from the Netherlands (21). However, long-term co-administration (especially over 6 months) would pose ischemic risk for patients with a low risk of GIB. Although PPI use was recommended for reducing bleeding while on DAPT in the latest guidelines, a definite duration of co-administration was not specified. It is hard for physicians to decide when to quit PPI use to ensure maximum benefit for patients. Our results provide some insight into this problem, and we derive that <6-month co-administration might be suitable.

## Limitation

There are some limitations in our manuscript: (1) Although CAMI is a large-scale and multicenter registry, our research is a retrospective study. Therefore, the two groups were not comparable to some extent. This would affect the assessment of the effect of PPIs on clinical outcomes. We have used statistical approaches (PSM or multiple regression) to diminish the bias. (2) CAMI could not evaluate the individual effect of PPIs on endpoints; we admitted that drug interaction between different PPIs and P2Y_12_ could affect the clinical events in patients and further better-designed research is warranted. (3) The CAMI project was launched in 2016, and clopidogrel was prevalent in that period because limited research and guidelines recommended other P2Y_12_. Further sub-analysis and studies evaluating the effect of PPIs on other P2Y_12_ inhibitors are warranted. (4) The inclusion of only the Chinese population might limit its applicability to other populations.

## Data Availability Statement

The original contributions presented in the study are included in the article/Supplementary Material, further inquiries can be directed to the corresponding author/s.

## Ethics Statement

The original study on which this paper is based was reviewed and approved by Ethics Committee of Fuwai Hospital. Reference Number: 2012-398. The patients/participants provided their written informed consent to participate in the original study.

## Author Contributions

WS was responsible for literature search, study design, data management, data interpretation, and writing. XF and LN was responsible for data analysis, data interpretation, and writing. JY, MeiY, and HY was involved in study design, statistical analysis plan, and data interpretation. MenY and YY were involved in data interpretation and writing of the manuscript. All authors contributed to the article and approved the submitted version.

## Funding

This work was supported by the CAMS Innovation Fund for Medical Sciences (CIFMS) (2016-I2M-1-009), Twelfth Five-Year Planning Project of the Scientific and Technological Department of China (2011BAI11B02), and National Natural Science Foundation of China (No. 81670415).

## Conflict of Interest

The authors declare that the research was conducted in the absence of any commercial or financial relationships that could be construed as a potential conflict of interest.

## Publisher's Note

All claims expressed in this article are solely those of the authors and do not necessarily represent those of their affiliated organizations, or those of the publisher, the editors and the reviewers. Any product that may be evaluated in this article, or claim that may be made by its manufacturer, is not guaranteed or endorsed by the publisher.
