# Supplementary material for: The Clinical Impact of Proton Pump Inhibitors When Co-Administered With Dual Antiplatelet Therapy in Patients Having Acute Myocardial Infarction With Low Risk of Gastrointestinal Bleeding: Insights From the China Acute Myocardial Infarction Registry
Source: Front Cardiovasc Med. 2021 Sep 22;8:685072. doi: 10.3389/fcvm.2021.685072 (PMC8492977; doi:10.3389/fcvm.2021.685072)
Supplement: Supplementary file 1 [file Table_1.DOCX]

**Supplementary**

**Table 1.** **Discrimination and calibration of the propensity score matching model**

|  | ROC analysis | | | Hosmer-Lemeshow Test | |
| --- | --- | --- | --- | --- | --- |
| Index | AUC | 95% CI | P value | Chi-Square | P value |
| PSM Model | 0.514 | (0.4713, 0.5981) | <0.0001 | 8.5133 | 0.372 |

Variables in the propensity score matching (PSM) model included age; female, hypertension; dyslipidemia; stroke; malignancy; STEMI; systolic BP; Killip class IV; Hct; Asprin; GPIIb/IIIa receptor inhibitor; oral anticoagulants; heparin/LMWH; statin; β-blockers; ACEI/ARB

Discrimination of the model was defined with area under the curve (AUC) by receiver-operating characteristic curve (ROC) analysis. AUC P value <0.05 was considered that the prediction was significant. Calibration quantified how close the predicted probabilities match the actual experience and it was assessed using Hosmer-Lemeshow good of fit test, in which Chi-square statistics were calculated and P>0.05 indicated the calibration was significant.

**Table 2: Baseline Clinical Data in Patients With and Without PPIs After PSM**

| **Variables** | **With PPIs**  **(n=5014)** | **Without PPIs**  **(n=5014)** | ***P*** |
| --- | --- | --- | --- |
| **Demographics** |  |  |  |
| **Age** | 57.21 ±11.44 | 57.51 ±11.34 | 0.6133 |
| **Female** | 1043(20.8%) | 1028(20.5%) | 0.5961 |
| **Medical history** |  |  |  |
| **Hypertension** | 2447(48.8%) | 2361(47.1%) | 0.1996 |
| **Dyslipidemia** | 361(7.2%) | 341(6.8%) | 0.0811 |
| **Diabetes mellitus** | 973 (19.4%) | 958 (19.1%) | 0.1449 |
| **Myocardial infarction** | 441 (8.8%) | 366 (7.3%) | 0.0876 |
| **PCI** | 227(4.5%) | 226(4.5%) | 0.9532 |
| **CABG** | 15(0.3%) | 15(0.3%) | 0.9415 |
| **Congestive heart failure** | 65(1.3%) | 65(1.3%) | 0.6917 |
| **Stroke** | 406(8.1%) | 361(7.2%) | 0.0612 |
| **Chronic kidney disease** | 45(0.9%) | 45(0.9%) | 0.8551 |
| **Malignancy** | 50(1.0%) | 40(0.8%) | 0.0544 |
| **Admission features** |  |  |  |
| **STEMI** | 3921(78.2%) | 3780(75.4%) | 0.0411 |
| **Heart rate (beats/min)** | 78.61 ±20.13 | 78.17 ±18.75 | 0.6192 |
| **Systolic BP (mmHg)** | 128.41±24.12 | 129.56±25.33 | 0.0730 |
| **Killip class IV** | 160(3.2%) | 140(2.8%) | 0.0471 |
| **Hb (g/L)** | 138.71±19.31 | 139.01±21.06 | 0.1934 |
| **Hct (%) (Q1;Q3)** | 41.31±59.88 | 39.81±14.13 | 0.0318 |
| **CRUSADE score** | 17.72±13.61 | 17.92±14.03 | 0.5433 |
| **Pre-hospital medications, n (%)** |  |  |  |
| **Asprin** | 481(9.6%) | 506(10.1%) | 0.0616 |
| **P2Y12 receptor inhibitor** | 150(3.0%) | 160(3.2%) | 0.2411 |
| **In-hospital medications, n (%)** |  |  |  |
| **GPIIb/IIIa receptor inhibitor** | 1715(34.2%) | 1429(28.5%) | 0.0129 |
| **Oral anticoagulants** | 50(1.0%) | 105(2.1%) | 0.0000 |
| **Heparin/LMWH** | 4703(93.8%) | 4522(90.2%) | 0.0017 |
| **Statin** | 1574(31.4%) | 1464(29.2%) | 0.0000 |
| **β-blockers** | 3710(74.0%) | 3575(71.3%) | 0.0000 |
| **ACEI/ARB** | 1895(37.8%) | 1960(39.1%) | 0.0271 |
| **Treatment, n (%)** |  |  |  |
| **Primary PCI** | 1835(36.6%) | 1860 (37.1%) | 0.0829 |
| **Primary CABG** | 15 (0.3%) | 10 (0.2%) | 0.7014 |
| **Thrombolysis** | 356 (7.1%) | 366 (7.3%) | 0.3014 |

Abbreviation: PSM= propensity score matching; PCI=percutaneous coronary intervention; CABG= coronary artery bypass grafting; STEMI=ST-elevation myocardial infarction; BP=blood pressure; Hb=hemoglobin; Hct=hematocrit; CRUSADE=can rapid risk stratification of unstable angina patients suppress adverse outcomes with early implementation of the ACC/AHA guidelines; GPIIb/IIIa=glycoprotein IIb/IIIa LMWH=low molecular weight heparin; ACEI/ARB=angiotensin converting enzyme inhibitor/angiotensin receptor blocker;

**Table 3:In-hospital, 1-year and 2-year results of Patients with and without PPIs after PSM**

| **Clinical endpoints** |  | **In hospital** | | |  | **1-year** | | |  | **2-year** | |
| --- | --- | --- | --- | --- | --- | --- | --- | --- | --- | --- | --- |
|  |  | **Adjusted OR (95% CI)** | | ***P***  **value** |  | **Adjusted HR (95% CI)** | ***P* value** | |  | **Adjusted HR (95% CI)** | ***P* value** |
| **GIB** |  | 5.314 (2.879-10.514) | <.0001 | |  | 4.127 (2.153-7.409) | | <.0001 |  | 3.491 (2.110-5.991) | <.0001 |
| **MACCE** |  | 0.996 (0.891-1.201) | 0.5216 | |  | 1.001 (0.924-1.223) | | 0.1512 |  | 1.103 (0.997-1.264) | 0.0614 |
| **All-cause death** |  | 0.912 (0.801-1.112) | 0.7002 | |  | 0.938 (0.806-1.081) | | 0.4103 |  | 0.963 (0.833-1.109) | 0.6844 |
| **MI** |  | 1.263(0.813-1.991) | 0.2113 | |  | **1.801 (1.283-2.512)** | | **0.0014** |  | **1.761 (1.221-2.400)** | **0.0002** |
| **Stroke** |  | **2.101(1.193-3.602)** | **0.0041** | |  | **2.254 (1.401-3.502)** | | **0.0033** |  | **2.018 (1.364-2.985)** | **0.0056** |

1. GIB=gastrointestinal bleeding; MACCE= major adverse cardiovascular and cerebrovascular events; MI=myocardial infarction; HR=hazard ratio
2. Variables included in the model: age; female, hypertension; dyslipidemia; stroke; malignancy; STEMI; systolic BP; Killip class IV; Hct; Asprin; GPIIb/IIIa receptor inhibitor; oral anticoagulants; heparin/LMWH; statin; β-blockers; ACEI/ARB;
